# Growth inhibition of *Acinetobacter* by 5-chloro-indole-3-acetic acid

**DOI:** 10.1128/spectrum.01858-25

**Published:** 2025-09-04

**Authors:** Tyler S. Laird, Alireza Gaeni, Johan H. J. Leveau

**Affiliations:** 1 Department of Plant Pathology, University of California Davis, Davis, California, USA; University of Manitoba, Winnipeg, Manitoba, Canada

**Keywords:** *Acinetobacter lactucae*, Acb complex, ESKAPE, *Acinetobacter baumannii*

## Abstract

The *Acinetobacter calcoaceticus-baumannii* complex includes high-priority, multidrug-resistant pathogens for which novel antibiotics are urgently needed. Many bacterial strains from this complex harbor a so-called *iac* gene cluster that codes for the catabolism of indole-3-acetic acid (IAA). Here, we demonstrate that possession and expression of *iac* genes represent an Achilles’ heel for *Acinetobacter* species, which can be exploited to suppress bacterial growth by treatment with IAA and its analog 5-chloro-IAA.

**IMPORTANCE:**

*Acinetobacter baumannii* is a deadly bacterial pathogen and one of the leading causes of hospital-acquired infections worldwide. It is also known for its resistance to many antibiotics currently available. In this study, we show that *Acinetobacter* bacteria choke on a mixture of IAA and 5-chloro-IAA, offering a path to the discovery and development of a novel drug treatment.

## OBSERVATION

Bacteria of the *Acinetobacter calcoaceticus-baumannii* (Acb) complex (1) are a leading cause of hospital-acquired urinary tract and bloodstream infections (2, 3). As a high-priority ESKAPE pathogen (4, 5), *A. baumannii* has an exceptional ability to develop resistance against a wide range of antibiotics (6, 7), which is of great clinical concern and highlights the urgent need for new types of drugs to treat *Acinetobacter* infections (8).

Many members of the Acb complex harbor a so-called *iac* gene cluster on their genome, which enables them to catabolize indole-3-acetic acid (IAA) (9). The *iac* gene cluster was first characterized in the plant leaf-associated bacterial strain *Pseudomonas putida* 1290 (10). The cluster codes for the enzymatic conversion of IAA to catechol, which is further metabolized by the *cat* gene products as part of the 3-oxoadipate pathway (11). In *A. baumannii* ATCC 19606, the *iac* gene cluster has been well studied (12, 13). This strain was originally isolated from the urine of a patient with a urinary tract infection (14). Human urine contains low concentrations of IAA (15), and it is therefore possible that the possession of *iac* genes and the ability to use IAA as a source of carbon and energy benefits *A. baumannii* as it colonizes the urinary tract. Indeed, several omics-based studies have shown expression of *iac* genes by *Acinetobacter* during conditions that mimic human infection (16–18). Thus, the *iac* gene-encoded pathway may be a promising target for treating *Acinetobacter* infections.

We hypothesized that the *iac*-encoded enzymes that convert IAA to catechol are more promiscuous than the *cat*-encoded enzymes that are responsible for the metabolism of catechol. In other words, we expected that *iac*-encoded enzymes will accept and process substituted IAAs (e.g., chlorinated IAA) into substituted catechols (e.g., chlorinated catechols), but that the *cat*-encoded enzymes will not be able to deal with such substituted catechols. This would lead to the accumulation of substituted catechols to high and toxic levels or in the formation of dead-end products such as protoanemonin, a compound that has antibacterial activity (19). As such, substituted IAAs might have the potential to act as antibiotics specifically active against *iac*-carrying bacteria, including *A. baumannii*.

We tested this hypothesis using *Acinetobacter lactucae*, formerly *A. dijkshoorniae* (20) as a proxy, more specifically the type strain of the species, NRRL B-41902^T^ (B-41902 for short). B-41902 belongs to the Acb complex, as does *A. baumanni* ATCC 19606 (3). Interestingly, B-41902 was originally isolated from plant leaves (21), as was the original *iac* strain *P. putida* 1290 (10). However, many other *A. lactucae* (*A. dijkshoorniae*) isolates have originated from clinical samples (22).

**Fig 1:**
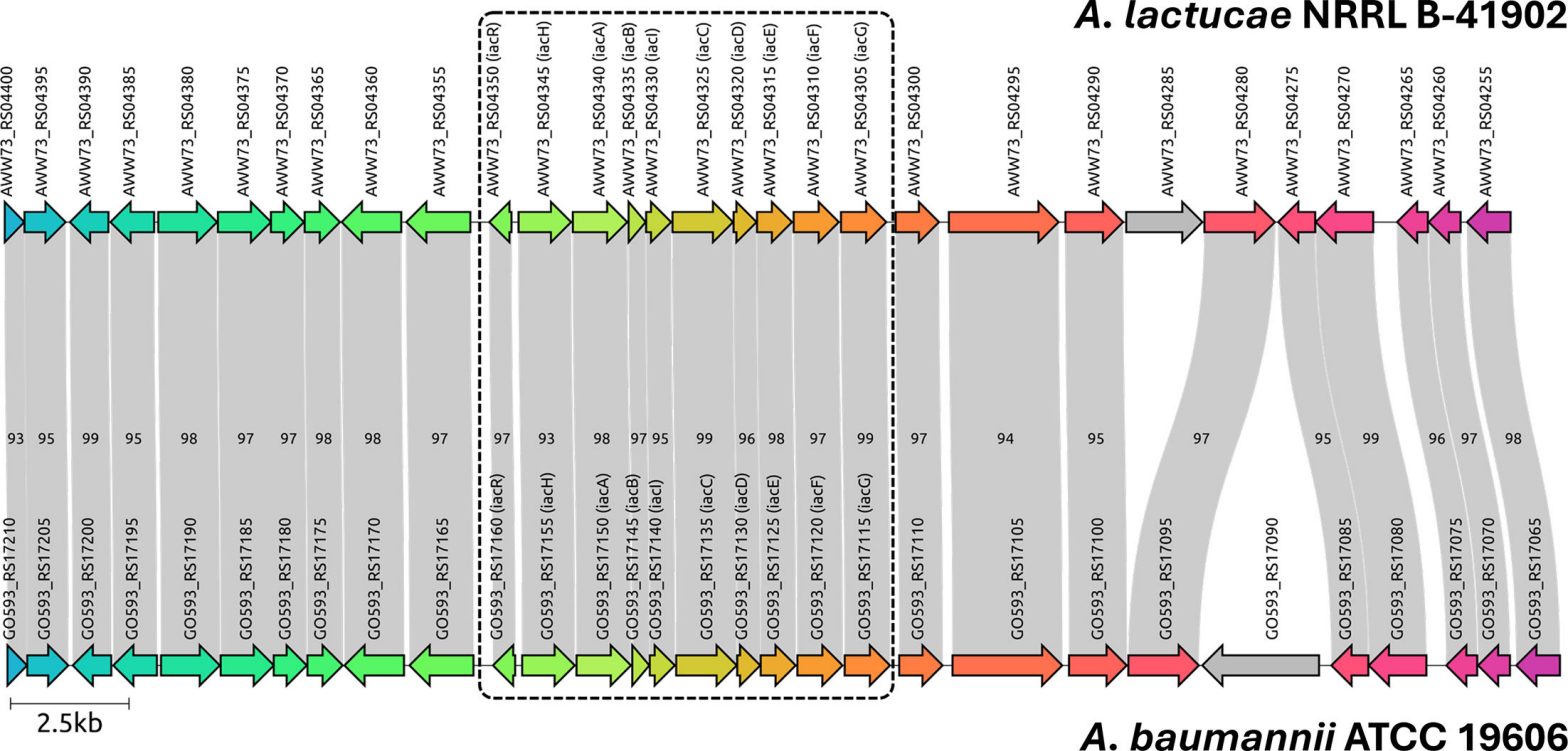
Alignment of *iac* gene neighborhoods from *A. lactucae* NRRL B-41902 (top) and *A. baumannii* ATCC 19606 (bottom). To generate this figure, the genomes of the two strains were retrieved as .gbff files from NCBI using accession numbers NZ_LRPE01000009.1 and NZ_CP046654.1, respectively, and aligned in clinker (23). Individual genes are labeled with their RefSeq locus tag. Gray ribbons connect orthologous genes; the number that is displayed on each ribbon refers to the percentage of amino acid identity shared between the predicted proteins of each gene pair. The dashed rectangle marks the *iac* gene cluster (*iacR-iacHABICDEFG*).

**Fig 2:**
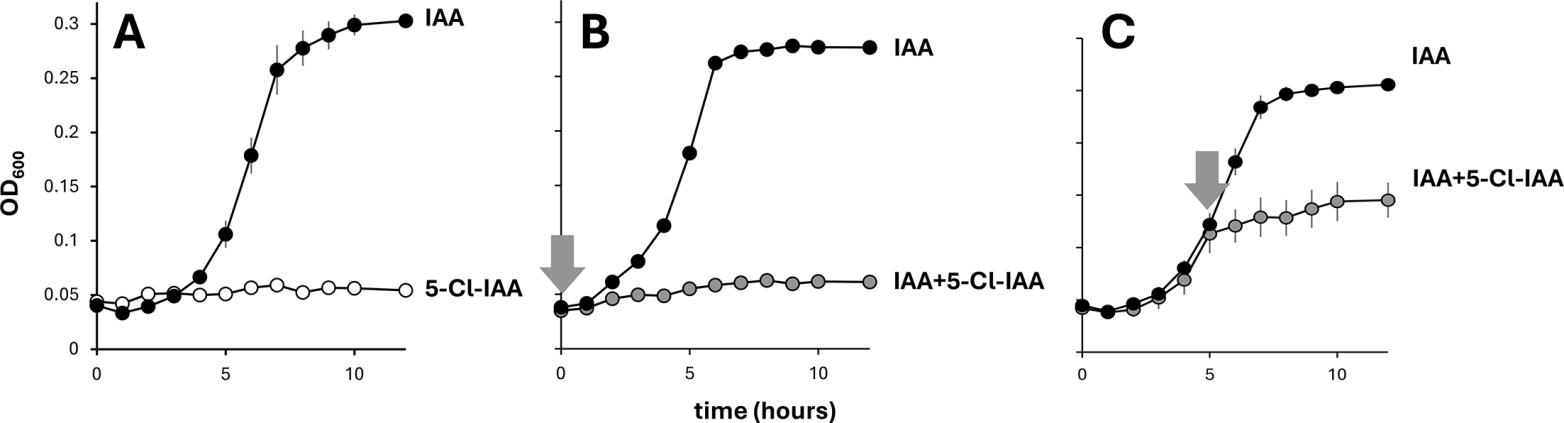
Growth of *A. lactucae* NRRL B-41902 on M9 minimal medium with added trace elements (Panke et al. [24]) and supplemented with either 1 mM IAA (black circles, **A, B and C**), with 1 mM 5-CI-IAA (white circles, **A**) or with a mixture of IAA and 5-Cl-IAA (gray circles), where 5-Cl-IAA was added either at the same time as IAA (**B**) or with a 6-h delay (**C**). Cells were inoculated from an overnight, washed culture on King’s Medium B Broth and incubated while shaking at 28°C. The optical density at 600 nm (OD_600_) was measured in a GENESYS 50 spectrophotometer (Thermo Fisher). IAA and 5-Cl-IAA were sourced from Biosynth International. Shown are the averages of three replicated experiments, with the bars representing the standard error.

We identified a complete *iac* gene cluster in the genome sequence of *A. lactucae* NRRL B-41902 (Fig. 1). Alignment of this cluster and its gene neighborhood to the genome of *A. baumannii* ATCC 19606 revealed a high degree of gene synteny and coding similarity (Fig. 1). We confirmed that *A. lactucae* NRRL B-41902 (obtained from the ARS Culture Collection at the USDA; https://nrrl.ncaur.usda.gov) can use IAA but not 5-chloro-IAA (5-Cl-IAA) as a sole source of carbon and energy (Fig. 2A). When IAA and 5-Cl-IAA were offered as a mixture, growth of B-41902 was severely compromised (Fig. 2B). Moreover, adding 5-Cl-IAA to a culture already growing on IAA caused growth to halt during the mid-exponential phase (Fig. 2C). This antibiotic effect of 5-Cl-IAA was specific for growth on IAA: the ability of *A. lactucae* NRRL B-41902 to grow on benzoate was not impacted by the addition of 5-Cl-IAA (Fig. S1).

Our findings suggest that the growth inhibitory effect of 5-Cl-IAA depends on expression of the *iac*-encoded pathway for IAA degradation. Previous observations show that *iac* genes are actively transcribed by *A. baumannii* during infection and are regulated by the same elements that control virulence (9). These findings invite a closer look at the use of 5-Cl-IAA (and possibly other IAA analogs, alone or in combination with IAA) as a promising addition to the urgently needed arsenal of new therapeutics against *A. baumannii*, which includes novel antibiotics (25, 26), mRNA vaccines (27), and bacteriophages (28). It is also of considerable interest that several other hospital-acquired and/or multidrug-resistant ESKAPE pathogens possess an *iac* gene cluster, for example, *Klebsiella pneumoniae* and *Enterobacter* species (9). This suggests that our findings for exploiting the *iac* gene cluster as a therapeutic target could have impact and utility beyond just *A. baumannii*.

## SUPPLEMENTAL MATERIAL

Figure S1 spectrum.01858-25-s0001.tif Impact of 5-Cl-IAA on the growth of *A. lactucae* NRRL B-41902 on IAA or benzoate.

ASM does not own the copyrights to Supplemental Material that may be linked to, or accessed through, an article. The authors have granted ASM a non-exclusive, world-wide license to publish the Supplemental Material files. Please contact the corresponding author directly for reuse.
