# Supplementary figures and images for: Growth inhibition of Acinetobacter by 5-chloro-indole-3-acetic acid
Source: Microbiol Spectr. 2025 Sep 4;13(10):e01858-25. doi: 10.1128/spectrum.01858-25 (PMC12502551; doi:10.1128/spectrum.01858-25)

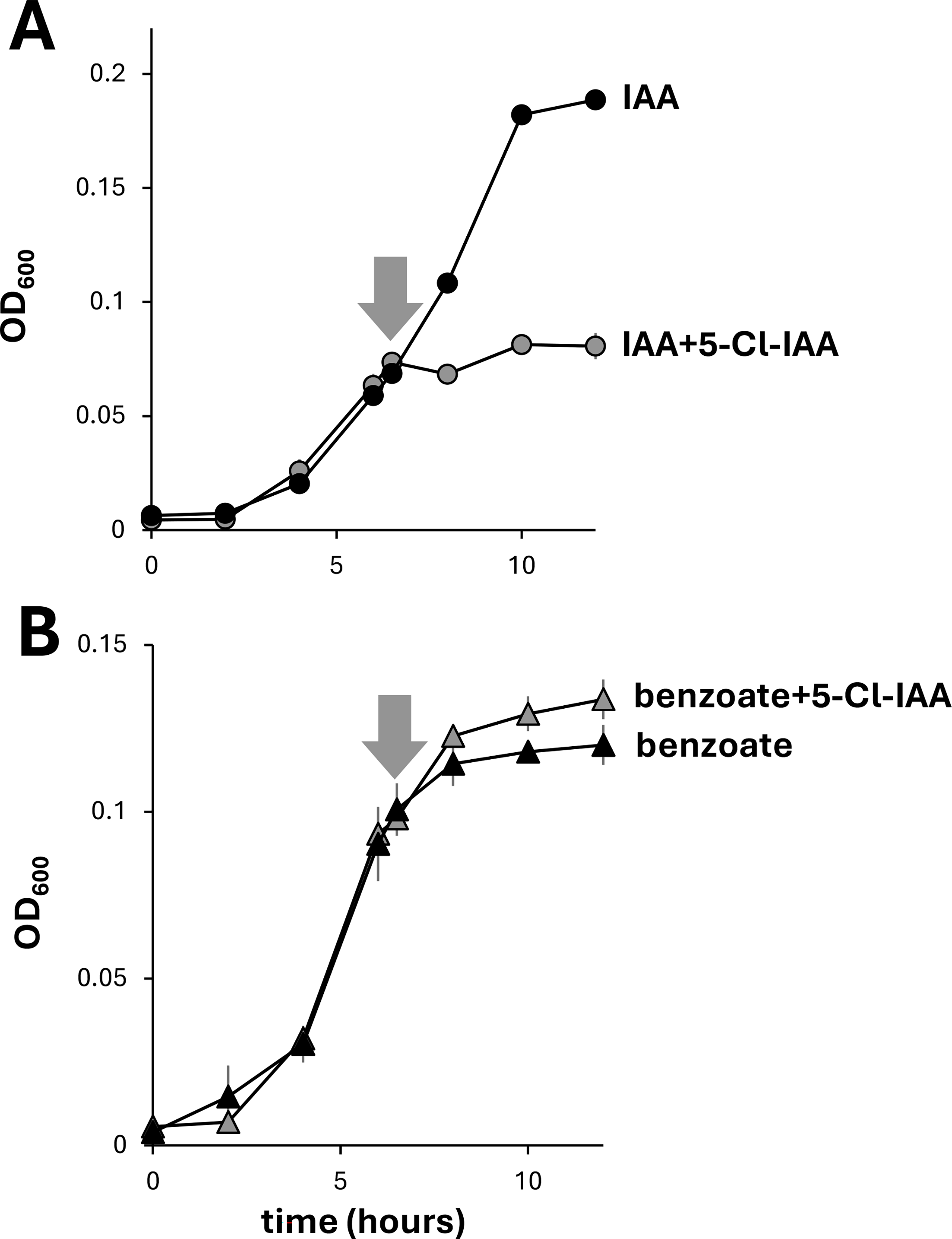

Supplement: Figure S1 — Impact of 5-Cl-IAA on the growth of A. lactucae NRRL B-41902 on IAA or benzoate. [file spectrum.01858-25-s0001.tif]
